# Clinical features and management of children with dengue-associated obstructive shock syndrome: A case report

**DOI:** 10.1097/MD.0000000000031322

**Published:** 2022-10-28

**Authors:** Thanh Tat Nguyen, Nhung Thi-Hong Le, Ngoc Minh Nguyen, Viet Chau Do, Tung Huu Trinh, Luan Thanh Vo

**Affiliations:** a Department of Infectious Diseases, Children’s Hospital No.2, Ho Chi Minh City, Vietnam; b Woolcock Institute of Medical Research, Ho Chi Minh City, Vietnam; c Pediatric Department, Binh Duong General Hospital, Binh Duong, Vietnam.

**Keywords:** clinical management, dengue obstructive shock syndrome, plasma leakage, point-of-care ultrasound, Vietnam

## Abstract

Dengue obstructive shock syndrome is a fatal complication commonly observed in the late critical phase of dengue infection and is associated with a high mortality rate. The main pathogenesis involves a dramatic increase in chest pressure, owing to severe plasma leakage and mechanical respiratory support, hampering the heart’s ability to pump effectively and impeding adequate blood venous return to the heart chambers. To date, there is a paucity of clinical data about Dengue obstructive shock syndrome reported.

**Patient concerns:**

The 2 reported patients presented with prolonged and decompensated dengue shock with critical multi-organ failures and mechanical ventilation. The patients’ hemodynamics were profoundly affected by high pressure in the thoracic and abdominal cavities resulting from Dengue-induced severe plasma leakage and mechanical ventilation.

**Diagnoses:**

Clinical presentations, laboratory data, mini-fluid challenge test, and point-of-care (POCUS) were used to make diagnoses and guide management.

**Interventions:**

Clinical monitoring, judicious fluid (colloids and blood products) administration guided by repeated POCUS to properly assess the adequacy of the intravascular volume, homeostasis adjustments by plasma exchange, and continuous renal replacement therapies.

**Outcomes:**

The patients had favorable outcomes.

**Lessons:**

Our study highlights the clinical manifestations and management of children with dengue obstructive shock syndrome and underscores the importance of monitoring hemodynamics by consecutive POCUS at the bedside in order to make a timely diagnosis and assess intravascular fluid volume inadequacy accurately as well as closely monitor the fluid management responses.

## 1. Introduction

Dengue infection, a common mosquito-borne disease in tropical regions, affects an estimated 2.5 billion inhabitants in more than 120 countries, with an estimated annual incidence of 390 million cases, half a million requiring hospital admission, and 22,000 global deaths each year.^[1,2]^ Dengue shock syndrome is a severe and fatal complication of dengue infection, and the dengue case-fatality rate in Asian populations ranges from 0.5% to 3.5%.^[3]^ Kirawittaya et al reported low intravascular blood volume, decreased blood flow into the heart, and reduced wall motion of the left ventricle in the diastolic period in critical dengue-infected pediatric patients with severe plasma leakage were significantly observed.^[4]^ Dengue obstructive shock syndrome is a fatal complication commonly observed in the very late critical phase of severe dengue patients with prolonged and decompensated shock, and it is associated with a high mortality rate despite intensive treatment. This syndrome is caused by a dramatic increase in thoracic pressure, which hampers the heart’s ability to pump effectively and impedes adequate venous return to the heart in dengue-infected patients with progressively severe plasma leakage (substantial fluid accumulation in the pericardial, pleural, and abdominal cavities) and mechanical respiratory support. Remarkably, point-of-care ultrasound (POCUS) plays a critical role in rapidly assessing cardiac systolic and diastolic dysfunction, intravascular volume status, pericardial-pleural effusions, and ascites; therefore, it is performed in dengue patients with tension-obstructive shock syndrome.^[4,5]^ Most importantly, judicious fluid therapy and the risk of pulmonary edema must be carefully considered, and colloid solutions are recommended for severe dengue shock syndrome.^[6,7]^ To date, there is a paucity of clinical data about dengue obstructive shock syndrome reported.^[4]^ Here, we describe 2 children with dengue obstructive shock syndrome at the pediatric intensive care unit, Children’s Hospital No.2 in Ho Chi Minh City, Vietnam, to add to the limited knowledge on the clinical characteristics and management of these patients.

## 2. Case reports

### 2.1. Patient 1

**Figure 1.**
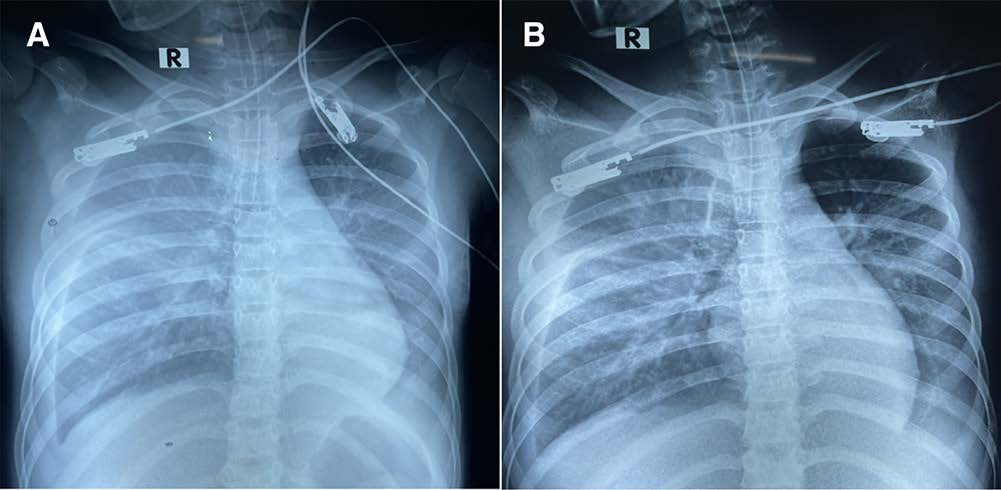
Chest X-rays showing bilateral pulmonary interstitial congestion with relatively small size of cardiac silhouette at tension-obstructive shock diagnosis (A) and the improvement in pulmonary congestion after the intensive shock management (B).

**Figure 2.**
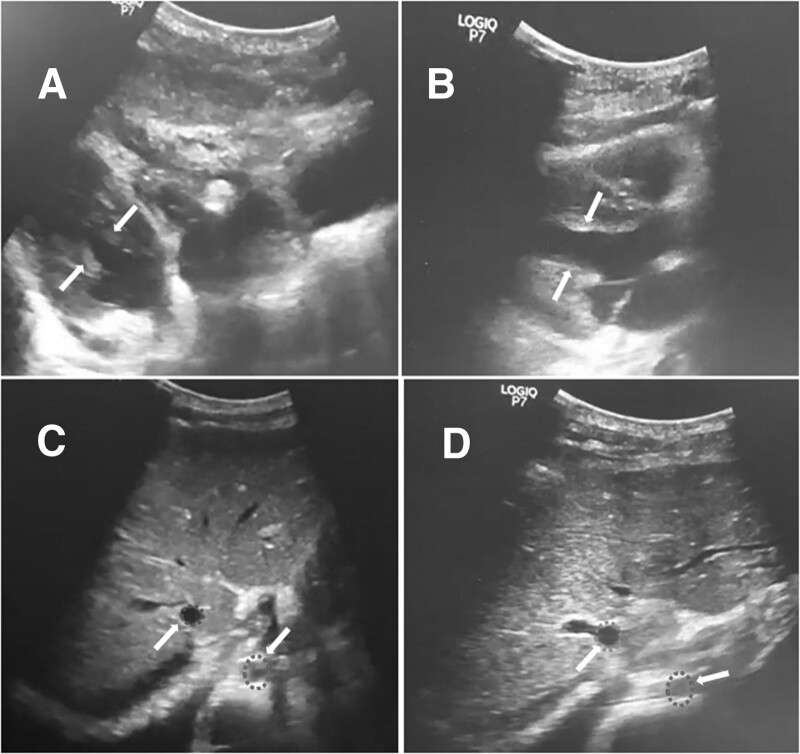
Point-of-care ultrasounds reveal mild pericardial effusion, significantly empty left and right ventricles, small end-diastolic volume of left ventricle (arrow lines), hyperdynamic heart and ejection fraction (EF) 50% (A), and substantial distention of the inferior vena cava (IVC) and IVC’s size (blue-colored dotted circle) closely half as the juxtaposed descending aorta (red-colored dotted circle) (C) at dengue obstructive shock. After shock management, there were significant improvements in early diastolic wall motion of the left ventricle, enhanced end-diastolic volume of left ventricle (arrow lines) and EF 65% (B), well distention and less variability of the IVC (D).

A 13-year-old female from Binh Duong province, northwest of Ho Chi Minh City, presented in February 2022 with a history of 06 days of high fever. She was diagnosed with β-thalassemia major at a tertiary pediatric hospital in Ho Chi Minh City at the age of 07 years. She received periodic blood transfusions, as indicated by a hematologist. Her main symptoms included persistently high fever, malaise, loss of appetite, vomiting, dark-colored loose stool, and petechiae, and she was undergoing cyclic menstruation. On admission to the provincial hospital, she was alert, with critically pale skin and mucosa, hepatosplenomegaly, increased heart rate of 130 beats per minute, blood pressure of 90/65 mm Hg, normal heart and lung sounds, and a soft abdomen. Laboratory results revealed pancytopenia (white blood cells, 2.7 × 10^9^/L, hemoglobin 4.1 g/dL, hematocrit 17%; platelets, 86 × 10^9^/L), positive dengue NS1 antigen and IgM ELISA-based tests, elevated transaminase (aspartate transaminase [AST], 468 U/L and alanine transaminase [ALT], 170 U/L), serum albumin 35 g/L, normal coagulation function tests, serum creatinine 67 µmol/L, cardiac troponin I 191 pg/mL, and creatine kinase-MB 30 U/L. Her bedside POCUS revealed normal cardiac structures, no signs of cardiac effusion, and normal systolic and diastolic functions, with an ejection fraction (EF) of 70%. The patient was diagnosed with dengue hemorrhagic fever. She was initially treated with a nasal oxygen cannula, red blood cell transfusion, and judicious normal saline infusion, in accordance with the World Health Organization guidelines.^[6]^ Fifteen hours after hospitalization, her clinical status progressively worsened with significant breathing difficulty, bilateral decreases in lung sound, marked ascites, mild pericardial effusion, and EF 64% on bedside sonography. The monitored laboratory tests revealed a dramatic reduction in platelet count of 25 × 10^9^/L, hematocrit of 31%, substantially elevated transaminase levels (AST 1081 U/L and ALT 336 U/L), marked dysfunction of coagulation tests (prothrombin time 53%, activated partial thromboplastin time 47%, and international normalized ratio [INR] 1.52), and a significant increase in cardiac troponin I of 1933 pg/mL, indicating myocarditis. Therefore, she was managed with nasal continuous positive airway pressure, commenced with colloid solution (dextran) and vasopressor infusion (noradrenalin 0.1 µg/kg per minute). At 24 hours after admission, her respiratory status progressively deteriorated and the abdominal pressure increased significantly; therefore, mechanical ventilation was indicated and a positive end-expiratory pressure of 12 cmH_2_0 and inspiratory pressure of 19 cmH_2_0 were achieved. Chest radiography revealed bilateral pulmonary interstitial congestion and a relatively small cardiac silhouette (Fig. 1A), and the Repeated POCUS revealed substantial fluid accumulation in the pleural and abdominal cavities, mild pericardial effusion, significantly empty left and right ventricles, hyperdynamic heart, and an EF of 50% (Fig. 2A), substantial distention of the inferior vena cava (IVC), and IVC diameter was nearly half that of the juxtaposed descending aorta (Fig. 2C; Supplemental Digital Content 1, http://links.lww.com/MD/H760. Nevertheless, her clinical status abruptly deteriorated after 4 hours of mechanical ventilation, presenting with restlessness, cold clammy skin, rapid weak pulse (170 beats per minute), invasive arterial blood pressure of 83/68 mm Hg, and capillary refill time >3 seconds. At this time, repeat laboratory test revealed the hematocrit 25%, blood lactate 5.1 mmol/L, and the arterial blood gases showed pH 7.36, pCO_2_ 26 mm Hg, HCO_3_^-^ 15 mmol/L and base excess −9.4 (Table 1). The ascites was dramatic, and the intra-abdominal pressure was as high as 28 cmH_2_0. Dengue obstructive shock syndrome resulting from high pressure in the thoracic and abdominal cavities has been established. Two mini-fluid challenge tests with colloid solutions (5 mL/kg in 15 minutes) resulted in a significant increase in invasive arterial blood pressure (118/47 mm Hg) and a decrease in pulse rate (130 beats per minute), indicating low intravascular blood volume and decreased blood flow into the cardiac chambers. On this basis, dengue obstructive shock in this patient was managed with high-dose colloid fluid (10 mL/kg/h × 2 hour), then sustained at 05 mL/kg/h. In addition, peritoneal drainage was performed to decompress the intra-abdominal pressure, positive end-expiratory pressure was adjusted to 10 cmH_2_0 according to the decompressed intra-abdominal pressure, and the vasopressor dose was also slightly scaled up to achieve the target for abdominal perfusion pressure. As a result, the patient’s clinical status of shock improved dramatically, with a marked increase in peripheral perfusion, a strong pulse of 130 beats per minute, and a blood pressure of 100/60 mm Hg. Repeated chest radiography after shock management showed considerable mitigation of pulmonary congestion (Fig. 1B). In addition, further POCUS revealed significant improvement in the early diastolic wall motion of the left ventricle and an EF of 65% (Fig. 2B), good distention, and less variability in the IVC (Fig. 2D; Supplemental Digital Content 2, http://links.lww.com/MD/H761. However, she later developed severe acute kidney failure (anuria, serum creatinine 371 µmol/L, glomerular filtration rate 11 mL/min/1.73 m^2^) and underwent continuous renal replacement therapy. The patient experienced gradual clinical improvement over 3 weeks and was discharged without complications.

**Table 1:** Clinical features, treatments, and outcomes of the patients with dengue obstructive shock syndrome in Vietnam.

Case	Age, sex	Comorbidity	Status of patients at the time of dengue obstructive shock	Radiological findings at shock	Status of patients after dengue obstructive shock management	Radiological findings after shock management	Treatments	Outcomes
1	13-yr-old, female	Thalassemia	Fever, vomiting, petechiae, critically pale skin, breathing difficulty.	Chest X-ray: Bilateral pulmonary congestion, small size of cardiac silhouette	Significantly clinical improvement: marked increase in peripheral perfusions, strong pulse 130 beats per minute and blood pressure 100/60 mm Hg	Chest X-ray: Considerable mitigation in pulmonary congestion	Mechanical ventilation, peritoneal drainage, colloid fluid infusion blood-product and albumin transfusions, vasopressors, inotrope continuous renal replacement therapy	Discharged, alive
						POCUS: Significant improvement in early diastolic wall motion of the left ventricle, EF 65%, well-distention and less variability of IVC		
				POCUS: Mild pericardial effusion, significantly empty left and right ventricles, hyperdynamic heart, EF 50%, substantial distention of the IVC and its size closely half as the juxtaposed descending aorta Dramatic fluid accumulation in pleural and abdominal cavities				
			Cold clammy skin, rapid weak pulse 170 bpm, blood pressure 83/68 mm Hg, CRT > 3 sec, bilateral decrease of lung sounds at 2 bases, substantial ascites Severe kidney failure (GFR = 11 mL/min/1.73 m^2^), intra-abdominal pressure = 28 cmH_2_O, HCT = 25%, PLT = 25 × 10^9^/L, AST = 1081 U/L, ALT = 336 U/L, Troponin I = 1933 pg/mL. Lactate = 5.1 mmol/L, NH3 = 130 µmol/L					
2	10-yr-old, male	Obesity	Comatose, HE grade 3, oxygenation support via endotracheal tube, mottled skin, rapid weak pulse 160 beats per minute, blood pressure 120/70 mm Hg, diffuse petechiae, bilateral decreased lung sound at 2 bases, substantial ascites, insidious nasal and oral bleedings, equal and reactive pupils HCT = 31%, PLT = 11 × 10^9^/L, AST = 13,860 U/L, ALT = 2451 U/L, Lactate = 6.3 mmol/L, NH3 = 201 µmol/L	Chest X-ray: Bilateral diffuse pulmonary congestion, small size of cardiac silhouette	Significantly clinical improvement:	Chest X-ray: Substantial improvement in pulmonary congestion	Mechanical ventilation, Colloid fluid infusion	Discharged, alive
					marked increase in peripheral perfusions, strong pulse 136 beats per minute and blood pressure 140/75 mm Hg, absence of pulmonary rales on auscultation			
							NaCl 3% infusion for cerebral edema treatment, Blood-product and albumin transfusions, Vasopressors Continuous renal replacement therapy Therapeutic plasma exchange therapy	
						POCUS: Significant improvement in early diastolic wall motion of the left ventricle, EF 67%, well-distention and less variability of the IVC		
				**POCUS**: Significantly empty left-and-right ventricles and hyperdynamic heart, absence of pericardial effusion, substantial distention of IVC, absence of the IVC’s respiration variation in size				

ALT = alanine aminotransferase (normal, <40), AST = aspartate aminotransferase (normal, <37), CRT = capillary refill time, EF = ejection fraction (%), GFR = glomerular filtration rate, HCT = hematocrit (%), HE = hepatic encephalopathy, IVC = inferior venous cava, POCUS = point-of-care ultrasound at the bedside.

### 2.2. Patient 2

A 10-year-old male with age-adjusted obesity (body mass index 24 kg/m^2^) from Binh Duong province, northwest of Ho Chi Minh City, presented in June 2021 with a 6-day history of high fever and a positive dengue NS1 antigen test. The patient was referred from a provincial pediatric hospital with a diagnosis of severe and prolonged dengue shock syndrome. On admission, he had a comatose status, hepatoencephalopathy grade 3, and was on oxygenation support via an endotracheal tube and mechanical ventilation, with a rapid weak pulse of 160 beats per minute, blood pressure of 120/70 mm Hg, mottled skin, diffuse petechiae, multiple bruises at injection sites, normal heart sounds and bilaterally decreased breathing sounds at 2 lung bases, substantial ascites, insidious nasal and oral bleeding, and equal and reactive pupils. Laboratory tests revealed a hematocrit of 25%, platelet count of 11 × 10^9^/L, critical transaminases (AST 3646 U/L and ALT 1122 U/L), serum albumin 14 g/L, marked dysfunction of coagulation tests (INR 2.8), and blood lactate of 2.9 mmol/L. Chest radiography revealed bilateral diffuse pulmonary interstitial congestion and a small cardiac silhouette. The total infused crystalloid fluid was 170 mL/kg in 48 hours. The patient was then managed with colloid solution (hydroxyethyl starch 6%) at 10 mL/kg/h for 2 hours, then sustained at 0.5 mL/kg/h and noradrenalin (0.1 µg/kg per minute), red blood cells, fresh frozen plasma and albumin transfusions, and infusion of saline solution (3%) for treating cerebral edema. POCUS revealed marked distention of the IVC and the absence of IVC respiration variation in size; nevertheless, the patient’s obesity hampered the performance of ultrasound. After 11 hours of hospitalization, the patient was still comatose and showed no marked improvement in dengue shock status, pulse of 145 beats per minute, blood pressure of 110/65 mm Hg, and oliguria. The further laboratory tests showed significant metabolic acidosis (pH 7.19, HCO_3_^-^ 10 mmol/L, base excess -18), blood lactate 6.3 mmol/L, serum ammonia (NH3) 201 μmol/L, dramatically elevated transaminases (AST 13,860 U/L and ALT 2451 U/L), severe dysfunction of coagulation tests (INR 3.23), cardiac troponin I 0.32 μg/L (Table 1). Repeated POCUS at the bedside revealed significantly empty left and right ventricles and a hyperdynamic heart, absence of pericardial effusion, significant distention of the IVC, and absence of IVC respiration variation in size. Based on these findings, the patient continued to be administered colloid solution, 15 mL/kg/h × 2 hours). As a result, the patient’s dengue shock status significantly improved, clinically manifesting as a dramatic increase in peripheral perfusion, strong pulse of 136 beats per minute, blood pressure of 140/75 mm Hg, and absence of pulmonary moist rales on auscultation. Chest radiography after shock management showed a substantial improvement in pulmonary congestion. In particular, further POCUS at the bedside revealed significant improvement in the early diastolic wall motion of the left ventricle, EF of 67%, good distention, and less variability of the IVC. However, the patient developed severe acute kidney failure (anuria, glomerular filtration rate 10 mL/min/1.73 m^2^). Therefore, continuous renal replacement therapies (2 weeks) and therapeutic plasma exchange (2 cycles) were administered to manage the severe liver and kidney failure. The patient experienced gradual clinical improvement over 07 days, completely recovered after 3 weeks and was discharged without complications.

## 3. Discussion

In this study, we present 2 pediatric patients who were previously managed with a large amount of fluid due to severe plasma leakage and required mechanical ventilation. This condition is rarely observed in contrast to its high mortality rate due to prolonged dengue shock accompanied by multi-organ failures; nevertheless, there is no treatment recommendation in dengue guidelines by the World Health Organization in 2009.^[6]^ Our study demonstrates several important clinical features and management of patients with dengue obstructive shock syndrome.

First, this condition can be observed in the late critical phase of dengue infection in patients with progressively severe plasma leakage and mechanical ventilation, and is associated with high mortality despite intensive treatment. The high increase in thoracic pressure dramatically impedes adequate venous return to the cardiac chambers and reduces the filling of the left and right ventricles at the end of the diastolic phase.^[8]^ In addition, drastically increased intra-abdominal pressure due to substantial ascites in severe dengue infection can obstruct the IVC, further decreasing blood volume to the heart chambers, as observed in Patient 1.^[9]^ In this regard, obesity, severe pleural effusion and ascites, mechanical respiratory support, and/or pericardial effusion are significant risk factors for developing DDES, as seen in 2 patients in this report. Most remarkably, Kirawittaya et al demonstrated that dengue-infected patients with severe plasma leakage commonly experience left ventricular systolic and diastolic dysfunction and reduction of left ventricle filling due to decreased movement of the left ventricle wall.^[4]^ These hemodynamic abnormalities and cardiac dysfunctions predispose patients with dengue to pulmonary edema and complicate fluid management and responses.^[4]^ As seen in patients 1 and 2 in this report, there was a substantial therapeutic conflict between the risk of pulmonary edema and absolute intravascular volume inadequacy due to progressive plasma leakage, clinically evidenced by positive responsiveness by mini-fluid challenge tests. Crucially, decreased movement of the left ventricle wall has been reported to have a transient effect;^[4]^ therefore, a significant reduction of left ventricle filling has a more profound effect on cardiac dysfunction in patients with severe dengue. In addition, positive mini-fluid challenge tests indicate that improving blood perfusion to internal organs and peripheral vessels is a higher priority than left-sided heart failure with preserved EF.^[8]^ This is supported by the relatively normal EF in patients 1 and 2 (EF ranging from 50% to 67%) despite left ventricular systolic and diastolic dysfunction on bedside sonography. On this basis, our therapeutic approach was to treat patients with Dengue obstructive shock syndrome with colloid solution infused from 10 to 20 mL/kg/h for 2 hours, subsequently tapering the dose of colloid fluid administration based on ventricular filling guided by POCUS, and closely monitoring tissue perfusion. Notably, colloid solutions were recommended for patients with severe and prolonged dengue shock syndrome on the basis of effective restoration of intravascular volume.^[6,7,10]^ However, excessive colloid fluid administration increases the risk of severe acute kidney injury, which may require continuous renal replacement therapy.^[11]^ We also supplemented patients with blood products to minimize colloid administration. Our therapeutic approach seems to be sensible, as observed in improving the survival outcomes of the 2 patients reported; nonetheless, further cohort studies are needed to elucidate this.

Second, comorbidities such as thalassemia, obesity, myocarditis, and severe liver and kidney failure, as observed in patients 1 and 2, will critically complicate the clinical management and outcomes of dengue patients with obstructive shock syndrome.^[12,13]^ Patient 1 experienced myocarditis with a significant elevation in cardiac troponin I; nevertheless, this abnormality was previously reported to have a transient effect and resolves spontaneously without specific treatment.^[4]^ These comorbidities dramatically decrease left ventricular systolic and diastolic dysfunctions, which critically aggravate shock status, leading to high mortality.^[12]^

In addition, we propose a case definition for dengue obstructive shock syndrome. Pediatric patients with dengue obstructive shock syndrome are defined as children with laboratory-confirmed dengue infection in the critical dengue phase with progressively severe plasma leakage presenting as substantial fluid accumulation in the pericardial, pleural, and abdominal cavities and edema. The patients are ongoingly stable with maintenance fluid management in accordance with the dengue treatment algorithm and mechanical respiratory support; however, they abruptly experience dramatic hypoperfusion of organs manifested as rapid weak pulse, significantly decreased blood pressure, mottled skin, cold clammy skin, and prolonged capillary refill time of >3 seconds.^[3]^ POCUS reveals significantly empty left and right ventricles, a hyperdynamic heart, and a plethoric IVC.^[4,5]^ This newly developed case definition will substantially aid timely clinical diagnosis and prompt appropriate intervention to enhance patient survival.

Another significant point is that POCUS is increasingly applicable in emergency and resuscitation units.^[14,15]^ POCUS is a powerful bedside tool for rapid diagnosis and severity prediction, guiding accurate fluid responsiveness to reduce mortality in critically ill patients.^[16,17]^ We performed consecutive POCUS in patients with severe dengue infection in the pediatric intensive care unit to examine fluid accumulation in the pleural and abdominal cavities, pericardial effusion, ventricular filling volume, systolic and diastolic dysfunctions, IVC distention, and the diameter ratio between the IVC and juxtaposed descending aorta (IVC/Ao).^[4]^ Lim et al showed that an IVC/Ao ratio <0.8 was statistically associated with hematocrit elevation and dengue shock syndrome in Dengue-infected children.^[18]^ Therefore, consecutive POCUS can be applied to patients with dengue obstructive shock to make a timely diagnosis and accurately assess fluid administration as well as to monitor fluid treatment responses.

This case study highlights the clinical manifestations and management of patients with dengue obstructive shock syndrome, underscoring the importance of hemodynamic monitoring by consecutive POCUS for appropriate fluid assessment and management. It also calls for clinical studies to determine the incidence, risk factors, and outcomes of this understudied complication in dengue patients with prolonged and decompensated shock.

## Author contributions

All authors have contributed to and approved the final manuscript.

**Conceptualization:** Luan Vo Thanh, Thanh Tat Nguyen.

**Funding acquisition:** Viet Chau Do.

**Investigation:** Luan Vo Thanh, Thanh Tat Nguyen, Nhung Thi-Hong Le.

**Methodology:** Luan Vo Thanh, Thanh Tat Nguyen.

**Supervision:** Ngoc Minh Nguyen, Viet Chau Do, Tung Huu Trinh.

**Writing – original draft:** Luan Vo Thanh, Thanh Tat Nguyen.

**Writing – review & editing:** Luan Vo Thanh, Thanh Tat Nguyen, Ngoc Minh Nguyen, Viet Chau Do, Tung Huu Trinh.

## Acknowledgments

We are grateful to the patients who participated in this study and gave us permission to report their details. We also thank Nguyen Tat Dat for preparing this manuscript for publication.

## Supplementary Material


